# Knowledge, Attitude, and Motivation Regarding Transcranial Direct Current Stimulation (tDCS) Among Rehabilitation Specialists in Saudi Arabia: A Cross-Sectional Study

**DOI:** 10.3390/healthcare13111300

**Published:** 2025-05-29

**Authors:** Alaa M. Albishi, Ahmed O. Alokaily, Madhawi K. Altaib, Mohammed F. Alharbi, Ahmed A. Aldohbeyb

**Affiliations:** 1 Department of Health Rehabilitation Sciences, College of Applied Medical Sciences, King Saud University, P.O. Box 10219, Riyadh 11433, Saudi Arabia; 2 Department of Biomedical Technology, College of Applied Medical Sciences, King Saud University, P.O. Box 10219, Riyadh 11433, Saudi Arabia; 3 Department of Speech-Language Pathology and Audiology, College of Medical Rehabilitation Sciences, Taibah University, Madina 42353, Saudi Arabia

**Keywords:** rehabilitation, knowledge, attitude, motivation, physical therapist, brain stimulation, tDCS, clinicians, Saudi Arabia

## Abstract

**Background:** Transcranial direct current stimulation (tDCS) has exhibited potential in augmenting motor functions, neuroplasticity, and cognitive abilities within neurological rehabilitation contexts. Despite these promising outcomes, the utilization of tDCS in both research and clinical environments in Saudi Arabia remains relatively unexplored. Objective: This study aims to evaluate the knowledge, attitudes, and motivations of rehabilitation specialists in Saudi Arabia concerning tDCS. **Methods:** A cross-sectional observational study was conducted through an online questionnaire, involving 112 registered rehabilitation professionals. **Results:** While 58% of respondents acknowledged tDCS as a therapeutic tool, the overall knowledge level was modest, with a mean score of 3.0 ± 2.7 out of 9. Enhanced levels of knowledge were associated with individuals holding doctoral degrees (*p* = 0.02) and those with international training exposure (*p* = 0.03). Despite the limited knowledge base, an overwhelming 94.64% of participants displayed a neutral to positive attitude towards tDCS, with 52.7% recognizing its potential efficacy in neurological recovery. Principal obstacles to implementation included safety-related concerns (21.4%) and the need for specialized training (23.3%). **Conclusions:** Rehabilitation specialists in Saudi Arabia generally hold a favorable view of tDCS and demonstrate an eagerness to pursue further training. The results underscore the imperative for enhanced educational initiatives and practical training to address knowledge deficiencies and facilitate the seamless integration of tDCS into clinical routines. The implementation of structured training programs could not only reinforce adoption rates but also optimize the role of tDCS within rehabilitation.

## 1. Introduction

Transcranial direct current stimulation (tDCS) is a non-invasive brain stimulation (NIBS) technique that modulates neuronal activity and induces functional changes in the brain [1,2]. By applying a low, continuous electrical current over the scalp, tDCS can regulate cortical excitability and alter brain activity, ultimately enhancing motor output [3,4,5]. Due to its non-invasive nature and the minimal adverse effects observed in previous studies [6,7], researchers have explored its potential benefits for individuals with neurological impairments [1,2,3,4,5,6,7]. Numerous studies have investigated the effects of tDCS on various neurological conditions, aiming to promote functional recovery and improve motor and cognitive abilities [8].

Research suggests that tDCS can enhance neuroplasticity, increase cortical excitability, and improve motor performance, particularly when combined with conventional rehabilitation techniques [9,10,11]. Additionally, tDCS has been explored as an adjunct therapy for conditions such as multiple sclerosis, Parkinson’s disease, and traumatic brain injury, showing promising results in terms of functional recovery and motor performance [12,13]. Beyond motor rehabilitation, tDCS has demonstrated the potential to enhance executive function, working memory, and attention in individuals with Alzheimer’s disease and mild cognitive impairment [14]. It has also been investigated as a cognitive rehabilitation tool for mental health disorders such as schizophrenia and depression, with studies suggesting that it may contribute to cognitive improvement and symptom reduction [15].

Despite its therapeutic potential, the mechanisms underlying tDCS’s effects are still being explored. The efficacy of tDCS depends on factors such as electrode placement, polarity, and stimulation parameters, which influence its impact on neural networks [4,5,16,17,18]. For example, placing the cathodal electrode over the contralateral hemisphere can induce subthreshold depolarization, promoting cortical excitation in M1. Conversely, anodal tDCS (a-tDCS) has been shown to enhance excitability in the ipsilesional hemisphere, correlating with improved upper limb function and daily activity performance in stroke patients [19,20,21]. Various tDCS protocols can be integrated with other rehabilitation approaches to optimize outcomes [4,8,16,17,18,22,23,24,25,26,27,28,29]. However, further research is needed to maximize its effectiveness to refine stimulation parameters, establish standardized protocols, evaluate long-term effects, and identify patient populations that respond best to tDCS [8,22,23,24,25,26,27,28,29].

Despite the growing body of evidence supporting tDCS, its clinical and research applications remain limited in Saudi Arabia. Studies suggest that a lack of awareness among practitioners may be a key barrier to its adoption [18,30,31,32,33,34]. While some efforts have been made to assess rehabilitation practitioners’ knowledge and attitudes toward transcranial magnetic stimulation (TMS) [33], little attention has been given to understanding their attitudes, motivations, and willingness to integrate tDCS into neurorehabilitation. Furthermore, research on TMS, which targets the motor cortex, is also scarce in the country [31,32,33,34,35]. Thus, this study aims to assess the knowledge, attitudes, and motivations of rehabilitation specialists in Saudi Arabia regarding the use of tDCS in clinical practice.

## 2. Materials and Methods

The survey instrument was a self-administered questionnaire that was structurally adapted from previous studies that evaluated psychiatrists’ and rehabilitation professionals’ attitudes and knowledge in Saudi Arabia regarding TMS [33,35]. However, this version was modified to assess knowledge and attitudes related to tDCS in rehabilitation, as well as assessing the rehabilitation specialists’ motivation for tDCS applications in rehabilitation programs.

### 2.1. Sample Size Calculation

The sample size for this study was determined using the following formula [36]:

$$n=\frac{Z^{2}\times P\left(1-P\right)}{d^{2}}$$

In line with previous research, the precision (d), expected prevalence (P), and confidence level were set at 0.6, 0.1, and 95% (Z = 1.96), respectively [33,35]. Based on these parameters, the required sample size was determined to be 93 participants. To account for a potential 5% dropout rate, the target sample size was adjusted to 98 participants. To further minimize the risk of incomplete data due to dropouts or missing responses, the final target was increased to 112 participants.

According to a recent study, approximately 2337 licensed rehabilitation professionals are practicing in Saudi Arabia [37]. The questionnaire was electronically distributed to around 250 registered rehabilitation therapists, resulting in 112 completed responses and a response rate of 44.8%, which exceeds that reported in a previous study [33]. All completed responses were included in the final analysis.

### 2.2. Data Collection

An anonymous online survey, hosted via Google Forms, was distributed to registered and licensed rehabilitation specialists through verified networks of rehabilitation specialists to assess their motivations, attitudes, and knowledge regarding transcranial direct current stimulation (tDCS). The survey was made accessible online, with invitations sent through WhatsApp messages and emails to rehabilitation professionals affiliated with universities, public and private hospitals, and research institutions. A total of 112 licensed rehabilitation therapists working in Saudi Arabia participated in the study. The research was approved by Taibah University’s Internal Review Board (reference number CMR-ST-2023-06), and all participants provided informed consent before being enrolled in the study.

### 2.3. Questionnaire Characteristics

The 32-question survey was divided into four sections: general and demographic information (14 questions), knowledge (9 questions), attitude (5 questions), and motivation (4 questions). The demographic section collected data on each participant’s age, gender, level of education, subspecialty, years of work experience, number of conferences attended annually, workplace region, primary practice institution, types of neurological disorders among patients in their caseload, and preferred sources of information. Additionally, participants were asked whether they had received training abroad in the last six months, had experience using tDCS, believed they had sufficient knowledge of tDCS applications, and if tDCS was available at their workplace.

The knowledge section assessed participants’ understanding of the applications, procedures, and limitations of tDCS in rehabilitation. The attitude and motivation sections evaluated their perspectives and willingness to incorporate tDCS into practice.

### 2.4. Questionnaire Validation

Ten independent research experts with a postgraduate education from King Saud University and Taibah University reviewed the questionnaire to ensure content validity. Experts were selected based on their seniority and research experience, with a minimum of two years of experience. They were provided with clear evaluation guidelines, including the study objectives, survey domains (knowledge, attitude, and motivation), and related items. Each item was rated on a scale from 1 to 4, where “1” indicated irrelevance or lack of clarity and “4” indicated high relevance or clarity. Feedback from the experts was incorporated to improve the questionnaire’s clarity and face validity.

To quantify content validity, the experts’ relevance ratings were converted into binary values: scores of 1 or 2 were coded as 0 (not relevant), while scores of 3 or 4 were coded as 1 (relevant). The scale-level content validity index (S-CVI/Avg) was then calculated using the averaging method [38]. A minimum S-CVI/Avg score of 0.78 is considered acceptable for content validity. The revised survey achieved a content validity score of 0.98 for relevance and 0.99 for clarity across all domains, confirming its strong validity.

### 2.5. Data Analysis

IBM SPSS Statistics version 30 was used for data analysis. The scoring system, adapted from previous research, evaluated participants’ motivation, attitude, and knowledge of tDCS [33,35].

A binary scoring system was applied for the knowledge assessment, assigning one point for each correct response and zero for unclear or incorrect answers. The total knowledge score ranged from 0 to 9, with participants categorized into three levels: low (0–3), moderate (4–6), and high (7–9).

Attitude was measured using a five-point Likert scale, where 1 represented “strongly agree” and 5 represented “strongly disagree”, with a maximum possible score of 25 points. Negatively worded statements were reverse scored. Scores were classified as either positive (13–25) or negative (0–12). Items 3, 4, and 5 contained positive statements, while items 1 and 2 were negatively worded.

Descriptive statistics were used to summarize participants’ characteristics, responses, attitudes, and motivations, including means, frequencies, and standard deviations (SD). The normality of continuous variables (knowledge and attitude scores) was assessed using the Shapiro–Wilk test. Nonparametric tests were used since the distributions were skewed (*p* < 0.05).

The Kruskal–Wallis test was applied to analyze factors with more than two levels, such as region, primary practice institution, knowledge sources, annual number of conferences attended, subspecialty (general, neuro, orthopedic rehabilitation), and education level (bachelor’s, master’s, PhD). Post hoc analysis using the Mann–Whitney U test identified significant differences in knowledge and attitude scores. Binary variables, including gender, tDCS accessibility, tDCS experience, and overseas training, were also analyzed using the Mann–Whitney U test.

Spearman’s rank correlation was used to examine the relationship between participants’ age and their knowledge and attitude scores. A significance level of *p* < 0.05 was applied, and all results were reported as the mean ± SD.

## 3. Results

### 3.1. Participant Characteristics

The survey was completed by 112 rehabilitation specialists. The sample consisted of 72 participants (64.3%) female and 37 (35.7%) male participants, with an average age of 31.0 ± 6.1 years. Regarding education, 61.6% of participants held a bachelor’s degree in rehabilitation sciences, 24.1% had a master’s degree, and 14.3% had a doctorate.

The respondents represented a diverse range of rehabilitation subspecialties. The largest groups specialized in physical therapy (35.7%), orthopedic rehabilitation (16.1%), neurological rehabilitation (15.2%), and speech and swallowing therapy (15.2%). Smaller percentages were reported for pediatrics (9.8%), cardiopulmonary rehabilitation (4.5%), and occupational therapy (3.6%). Most participants attended at least one conference yearly (48.2%), while 39.3% attended two to three conferences, and 12.5% reported attending more than three conferences yearly. Moreover, only 26.8% reported having training programs abroad for more than six months.

Participants were from all regions of Saudi Arabia, with the highest representation from the central region (44.6%), followed by the western (21.4%), southern (13.4%), northern (11.6%), and eastern (8.9%) regions. Regarding sources of professional knowledge, 45.5% of participants primarily relied on scientific publications to stay updated. The majority of respondents practiced in general hospitals (44.6%), followed by those who worked in teaching hospitals (21.4%), private practice (13.4%), and specialized rehabilitation centers (8.9%). Additionally, 58% of our respondents reported having more than six years of working experience, while 21.4% have three to five years of working experience, and only 20.5% reported having two or fewer years of working experience.

The majority of respondents (81.4%) had no prior experience with tDCS. Moreover, 68.8% reported having minimal knowledge of tDCS applications, while 27.7% reported having medium knowledge, and only 3.6% self-reported an advanced level of tDCS applications. A significant proportion (85.7%) actively worked with patients with neurological disorders, with stroke rehabilitation being the most commonly treated condition, reported by 74.1% of respondents. Small percentages of participants reported being involved in the treatment of patients with movement disorders (7.1%), traumatic brain injuries (3.6%), and brain tumors (0.9%). Interestingly, 12.5% of participants indicated that they were not involved in treating neurological conditions.

Furthermore, only 12.5% of participants reported having access to tDCS systems at their workplace, while 18.6% indicated that they had previous experience with tDCS. A detailed breakdown of demographic data is presented in Table 1.

### 3.2. Assessment of tDCS Knowledge

The results from the knowledge section are summarized in Table 2. Over half of the participants (58.0%) were aware that tDCS could potentially be used as a treatment tool for neurological diseases. In comparison, only 10.7% knew it could not be used as a diagnostic tool in rehabilitation. Additionally, 41.1% correctly indicated that the number of tDCS treatment sessions varies depending on the case. However, the participants demonstrated limited knowledge of tDCS contraindications, such as epilepsy/seizures, febrile convulsions in infancy, and recurrent fainting spells, as well as the most common tDCS side effects.

Furthermore, a large proportion of the respondents did not know that tDCS could be utilized in different montages (54.5%), that it can enhance cortical excitability via the anodal montage (68.8%), or that it can induce a reduction in the cortical excitability via the cathodal stimulation paradigm (82.2%). The average knowledge score was 3.0 ± 2.7 SD, with only 33.9% of participants scoring above half of the maximum possible score. In sum, 58.9% of participants had low knowledge levels, 25.2% had moderate levels, and just 15.9% exhibited high levels of knowledge.

The average knowledge scores were 2.7 ± 2.7 (mean ± SD) for bachelor’s degree holders, 2.7 ± 2.5 for master’s degree holders, and 4.8 ± 2.6 for doctoral degree holders. Knowledge scores were compared across education levels (bachelor’s, master’s, and doctoral) to assess their impact on tDCS knowledge. A significant difference was found among the groups based on their education level (Kruskal–Wallis test: *p* = 0.023). The post hoc analysis revealed that the participants with doctoral degrees had significantly higher tDCS knowledge compared with those with bachelor’s degrees (Mann–Whitney U test: Z = −2.70, *p* = 0.007) and master’s degrees (Mann–Whitney U test: Z = −2.27, *p* = 0.024). However, no significant difference was observed between bachelor’s and master’s degree holders.

Moreover, rehabilitation specialists who self-reported having a medium or high level of knowledge about tDCS scored significantly higher than those who indicated that they had a low level of knowledge (low vs. medium: Mann–Whitney U test: Z = −4.46, *p* < 0.001 and low vs. high: Mann–Whitney U test: Z = −2.32, *p* = 0.021). Additionally, specialists who reported having trained abroad for more than six months had significantly higher knowledge scores than those who did not (Mann–Whitney U test: Z = −2.18, *p* = 0.029). In contrast, factors such as gender, region, main source of knowledge, primary practice institution, number of conferences attended annually, and accessibility to a tDCS system did not significantly affect knowledge scores. Additionally, no correlation was found between specialists’ knowledge and age.

### 3.3. Assessment of Attitude and Motivation Toward tDCS

The questionnaire items that assess rehabilitation professionals’ motivations and attitudes, as well as their response rate, are described in detail in Table 3 and Table 4. Regarding tDCS, the majority of participants had a neutral attitude. Nonetheless, 56.7% of respondents indicated interest in taking part in tDCS training programs offered by their institutions, and a noteworthy 52.7% recognized the potential of tDCS to improve neurological recovery when paired with conventional therapy.

On the other hand, just 12.5% of respondents thought that tDCS technology was unsafe as it is, while 24.1% pointed out that there are few data available on its use in rehabilitation. Additionally, just 26.8% of the rehabilitation therapists who took part said they would be prepared to suggest tDCS clinical studies to their patients. Out of a possible maximum score of 25, the average attitude score was 16.6 ± 2.7 (66.3%), indicating a generally positive attitude toward the use of tDCS in rehabilitation. Just 5.4% of specialists had a negative attitude, while the vast majority (94.6%) scored more than half of the potential attitude scores.

A statistical analysis was performed to investigate the variables affecting opinions about the use of tDCS in rehabilitation. According to the findings, there was no significant difference in the calculated attitude score across the following demographics: education level, gender, years of experience, system availability, workplace, training overseas, region, or stated knowledge level. Furthermore, there was no discernible relationship between participants’ ages and attitude scores.

The motivations of the rehabilitation professionals and their reservations about the use of tDCS were also evaluated through the questionnaire (see Table 4). In response to questions concerning the difficulties presented by current therapy programs for common neurological conditions, 50% of respondents cited treatment effectiveness as their top concern, followed by program length (25.7%) and program feasibility (22.3%). Of those who expressed concerns about incorporating tDCS into clinical practice, safety (21.4%) and training (23.3%) were the most frequently mentioned issues. In contrast, 28.9% of participants said they had no concerns at all. Regarding the level of improvement that they would need to see in their patients before incorporating tDCS into their therapy programs, the participants also differed; 24% said they would need to see more than a 50% enhancement in their patients before incorporating tDCS into their treatment plans, while 17.9% said they needed less than a 20% improvement.

### 3.4. Scale Reliability

The Cronbach’s alpha (α) reliability test was used to assess the internal consistency of the evaluation questionnaire. The reliability test results indicated that the knowledge section had a Cronbach’s α of 0.8, demonstrating good internal consistency. In contrast, the attitude section had a Cronbach’s α of 0.7, indicating acceptable internal consistency. The overall internal consistency of the questionnaire was 0.8, suggesting that it is a reliable measure of attitude and knowledge [39].

## 4. Discussion

The study utilized a self-administered survey to evaluate the rehabilitation professionals’ knowledge, attitudes, and motivations regarding tDCS. Despite variations in gender distribution, 64.3% of respondents were female, consistent with previous research [33,40], suggesting that rehabilitation practice in Saudi Arabia aligns with trends seen in other medical disciplines [41]. As was the case in earlier studies, the majority of participants (61.61%) held bachelor’s degrees, while 38% had advanced degrees, including doctorates [33,40]. The central region of Saudi Arabia had the highest concentration of respondents, likely due to the greater availability of hospitals and rehabilitation programs in that area [42,43]. Physical therapists comprised the majority of participants, whereas neurorehabilitation specialists represented a smaller percentage, reflecting a trend observed in previous research. The lower number of neurological specialists compared to orthopedic specialists may be attributed to healthcare system priorities and the greater emphasis on orthopedic rehabilitation in education [33,40,44,45].

### 4.1. Assessment of tDCS Knowledge Among Rehabilitation Specialists

An assessment of participants’ knowledge about tDCS revealed that more than half knew it could be used for neurological rehabilitation, aligning with substantial evidence supporting its effectiveness as a supplement to conventional therapies [1,2,3,4,5]. Most participants correctly identified tDCS as a therapeutic rather than a diagnostic tool, reflecting its widespread use as an intervention method. However, awareness regarding its diagnostic applications in rehabilitation was limited, possibly due to the role of physicians in diagnosing patients before referring them for rehabilitation interventions [46]. This finding is consistent with previous research suggesting that practitioners may recognize tDCS as a potential treatment option without fully understanding its applications [33]. Additionally, participants exhibited limited knowledge regarding tDCS montages, including their ability to increase cortical excitability via anodal stimulation and decrease excitability via cathodal stimulation. This lack of awareness may be due to the limited availability of tDCS research in Saudi Arabia and a general gap in education regarding the neurological and technical mechanisms of brain stimulation. Additionally, as brain stimulation courses are rarely included in undergraduate rehabilitation programs, this gap in knowledge may stem from educational limitations [33].

Our study also examined the effect of educational levels and training on our participants’ overall knowledge scores. Consistently with previous findings, respondents with PhD degrees scored significantly higher on knowledge assessments compared to those with bachelor’s degrees [33,40,47]. Additionally, participants who self-reported a medium or high level of tDCS knowledge performed significantly better in the knowledge assessments, mirroring prior studies demonstrating a correlation between perceived knowledge and actual performance [33]. Furthermore, specialists who had undergone over six months of international training had significantly higher knowledge scores, highlighting the importance of hands-on training in clinical interventions. Furthermore, we also examined the effect of equipment availability and experience utilizing tDCS on our participants’ overall knowledge scores. The majority of respondents lacked access to tDCS equipment and had no prior experience using it. This lack of exposure may explain their lower knowledge scores, as previous studies suggest that the availability of necessary equipment is a key facilitator in applying theoretical knowledge to practice [48,49]. Participants also demonstrated poor knowledge of common tDCS side effects and contraindications, including epilepsy, febrile seizures in infants, and recurrent fainting episodes. Inconsistencies in NIBS safety research and a lack of clear differentiation between absolute contraindications and conditions requiring caution may contribute to this knowledge gap [5,35,50,51].

### 4.2. Assessment of Attitudes Toward tDCS Among Rehabilitation Specialists

The study also explored rehabilitation specialists’ attitudes and motivations regarding tDCS. The results showed that the majority of participants had a positive attitude, with only 5.4% expressing opposing views. More than half of the respondents (52.7%) recognized tDCS’s potential to enhance neurological recovery when combined with conventional rehabilitation methods, and 56.7% were interested in attending institutional training programs. These findings indicate a growing interest in integrating NIBS into rehabilitation, aligning with previous studies examining rehabilitation professionals’ opinions about TMS [33]. Concerns about tDCS safety were minimal, with only 12.5% of participants expressing doubts about the technology’s current development stage. This is notable given their limited understanding of side effects and contraindications. The widespread acceptance of tDCS safety may be influenced by extensive research indicating minimal adverse effects [6,7,52] and the US Food and Drug Administration (FDA) classifying tDCS as a non-significant risk (NSR) technology [52,53]. Despite their lack of hands-on experience, participants expressed confidence in tDCS as a viable rehabilitation tool, suggesting enthusiasm for learning more and implementing it in practice.

### 4.3. Assessment of Motivation Toward tDCS Among Rehabilitation Specialists

The study also assessed motivations and concerns regarding tDCS adoption in clinical settings. Half of the respondents cited treatment effectiveness as a primary concern, followed by program length (25.7%) and program feasibility (22.3%). These concerns align with previous research suggesting that the length of rehabilitation stays may be suboptimal, which hinders maximum functional recovery [54]. Given tDCS’s potential to enhance rehabilitation outcomes and facilitate maximum recovery when combined with conventional rehabilitation paradigms, further studies are needed to determine the optimal dosage and duration for its use in clinical practice [4]. Meanwhile, concerns about incorporating tDCS into rehabilitation primarily revolved around safety (21.4%) and the need for specialized training (23.3%), emphasizing the importance of education and practical experience. Participants’ expectations for patient improvement before integrating tDCS into therapy varied; 24% required more than a 50% improvement, while 17.9% considered a 20% improvement sufficient. This discrepancy aligns with variations in tDCS effectiveness reported in prior studies, mainly when used as a standalone intervention [5,55]. These results also highlight participants’ lack of tDCS training and limited knowledge of its effects.

### 4.4. Study Strength

This study provides valuable insights into rehabilitation professionals’ knowledge and generally favorable attitudes toward tDCS, highlighting interest and knowledge gaps in its clinical use. One of the key strengths lies in emphasizing the importance of professional training and integrating tDCS-related topics into rehabilitation education, particularly those focusing on neuroplasticity principles in rehabilitation. This approach could enhance practitioners’ understanding of tDCS and its role as a complementary modality in modern rehabilitation. Ultimately, expanding tDCS education and training in Saudi Arabian clinical and research settings could improve patient outcomes.

### 4.5. Practical Implications

The findings of this study have important implications for clinical education. There is a clear need to incorporate tDCS into rehabilitation training curricula to address existing knowledge gaps and better prepare clinicians to integrate this technology into practice. In Saudi Arabia, targeted training could bridge the gap between research and clinical application, supporting the use of multimodal rehabilitation approaches that combine tDCS with conventional therapies. Expanding professional development opportunities would also promote evidence-based practice and strengthen the role of tDCS in clinical research. Building rehabilitation specialists’ expertise could drive the wider adoption of tDCS and accelerate the development of regulatory frameworks. As seen in countries including Brazil, Italy, and Germany, clinician training has been key to improving the access to and regulation of tDCS [53]. For example, in Brazil, the national organization for occupational and physical therapists has recommended tDCS, authorizing rehabilitation therapists to apply it alongside physical therapy to manage pain, enhance sensorimotor function, and support cognitive recovery [53]. With better training and awareness, Saudi Arabia could follow a similar path, helping to ensure that tDCS is used safely and effectively in clinical research.

### 4.6. Study Limitations and Future Research Directions

This study has several limitations. First, the survey was administered via Google Forms, requiring participants to use a computer-based platform, which may have restricted participation. Additionally, most respondents were from Saudi Arabia’s central region, limiting the generalizability of the findings. Another limitation of this study is the relatively low response rate. The survey was distributed to approximately 11% of all registered rehabilitation professionals in Saudi Arabia, with responses obtained from only about 5% of the total population of physical therapists in the country. This limited reach may affect the generalizability of the findings and should be considered when interpreting our conclusions about the knowledge, attitudes, and motivations regarding tDCS. Moreover, the study did not focus on specific rehabilitation subspecialties, reducing the specificity of the results. Future studies could target neurological specialists to assess their perspectives on and knowledge of tDCS in greater detail. Furthermore, this study focused on rehabilitation professionals, excluding other medical professionals and neuroscientists who may also benefit from tDCS applications. Future research should include a wider range of healthcare professionals to better understand clinical attitudes and expertise. Expanding the study to different medical fields would provide a more comprehensive perspective on tDCS implementation in clinical practice.

## 5. Conclusions

The study provides valuable insights into the attitudes and knowledge of Saudi Arabian rehabilitation specialists regarding tDCS. Our findings indicate that rehabilitation therapists in Saudi Arabia generally have a favorable attitude toward tDCS and possess a moderate level of knowledge about its applications. However, safety concerns and a lack of specialized training may present barriers to its adoption in clinical practice. Additionally, the results highlight that training and educational background significantly impact knowledge levels, emphasizing the need for structured education and hands-on training opportunities. To promote the integration of tDCS into rehabilitation practice, it is essential to address knowledge gaps, encourage positive attitudes, and support continuous professional development, ultimately enhancing clinical applications and improving patient functional recovery across various rehabilitation settings.

## Figures and Tables

**Table 1:** Demographic factors.

Item		N (%)
Age	Mean ± SD: 31.4 ± 6.1 SD	
Gender	Female	72 (64.3)
	Male	40 (35.7)
Education	Doctoral	16 (14.3)
	Master’s	27 (24.1)
	Bachelor’s	69 (61.6)
Rehabilitation subspecialty	Physical therapy	40 (35.7)
	Neurorehabilitation	17 (15.2)
	Pediatrics	11 (9.8)
	Orthopedic	18 (16.1)
	Occupational therapy	4 (3.6)
	Speech and swallowing therapy	17 (15.2)
	Cardiopulmonary therapy	5 (4.5)
Number of conferences attended annually	1	54 (48.2)
	2–3	44 (39.3)
	More than 3	14 (12.5)
Region	Central	50 (44.6)
	Western	24 (21.4)
	Eastern	10 (8.9)
	Northern	13 (11.6)
	Southern	15 (13.4)
Primary practice institution	General Hospital	50 (44.6)
	Teaching Hospital	24 (21.4)
	Private Practice	15 (13.4)
	Rehabilitation Center	10 (8.9)
	University	13 (11.6)
Years of experience	0–2	23 (20.5)
	3–5	24 (21.4)
	6–10	42 (37.5)
	>10	23 (20.5)
The main source of knowledge	Articles	51 (45.5)
	Conferences	12 (10.7)
	Textbooks	17 (15.2)
	Discussions with colleagues	25 (22.3)
	Personal experience with tDCS	7 (6.3)
Have you trained abroad for more than six months?	Yes	30 (26.8)
	No	82 (73.2)
Do you have a tDCS system at your workplace?	Yes	14 (12.5)
	No	98 (87.5)
What type of neurological cases do you most frequently encounter in your caseload	Stroke	83 (74.1)
	Traumatic brain injuries	4 (3.6)
	Movements disorders	8 (7.1)
	Brain tumors	1 (0.9)
	I do not see patients with neurological disorders	16 (14.3)
Do you have previous experience with a tDCS system?	Yes	19 (18.6)
	No	83 (81.4)
How would you assess your level of knowledge regarding tDCS?	Low	77 (68.8)
	Medium	31 (27.7)
	High	4 (3.6)

**Table 2:** Knowledge of tDCS among rehabilitation specialists.

Questions	Frequency (Percentage) of Responders		Mean ± SD
	Correct	Incorrect	
1. tDCS can be a diagnostic tool. (F)	12 (%10.7)	100 (%89.3)	0.1 ± 0.3
2. tDCS can be a treatment tool. (T)	65 (%58.0)	47 (%42.0)	0.6 ± 0.5
3. The recommended number of tDCS sessions varies according to the subject condition. (T)	46 (%41.1)	66 (%58.9)	0.4 ± 0.5
4. tDCS has different modes. (T)	51 (%45.5)	61 (%54.5)	0.5 ± 0.5
5. Anodal tDCS increases the excitability of the stimulated area. (T)	35 (%31.3)	77 (%68.8)	0.3 ± 0.5
6. Cathodal tDCS decreases the excitability of the stimulated area. (T)	20 (%17.9)	92 (%82.1)	0.2 ± 0.4
7. tDCS cannot be used in cases of epilepsy/seizures, febrile convulsions in infancy, and recurrent fainting spells. (T)	35 (%31.3)	77 (%68.8)	0.3 ± 0.5
8. tDCS can cause minor side effects such as a tingling and itching sensation. (T)	37 (%33.0)	75 (%67.0)	0.3 ± 0.5
9. tDCS can cause severe brain damage. (F)	32 (%28.6)	80 (%71.4)	0.3 ± 0.5
Total Knowledge Score			3.0 ± 2.7

**Table 3:** Attitude toward tDCS among rehabilitation specialists.

Questions	Frequency (Percentage) of Responders					Mean ± SD
	Strongly Agree	Agree	Neutral	Disagree	Strongly Disagree	
1. I believe that tDCS technology is risky.	2 (1.8)	12 (10.7)	60 (53.6)	32 (28.6)	6 (5.4)	3.3 ± 0.8
2. There is no sufficient evidence of the efficacy of tDCS in neurorehabilitation.	5 (4.5)	22 (19.6)	66 (58.9)	16 (14.3)	3 (2.7)	2.9 ± 0.8
3. tDCS should be combined with other conventional treatments to augment the patient’s recovery.	15 (13.4)	44 (39.3)	49 (43.8)	2 (1.8)	2 (1.8)	3.6 ± 0.8
4. I would like to participate in tDCS training within my institute.	18 (16.1)	45 (40.2)	35 (31.3)	10 (8.9)	4 (3.6)	3.6 ± 1.0
5. I recommend that my patients participate in tDCS clinical trials.	9 (8.0)	21 (18.8)	72 (64.3)	8 (7.1)	2 (1.8)	3.2 ± 0.8
Total Attitude Score		16.6 ± 2.7				

**Table 4:** Motivations regarding tDCS among rehabilitation specialists.

Item		N (%)
Do you have any concerns regarding the integration of tDCS into your practice?	Safety	24 (21.4)
	Cost	8 (7.1)
	Administrative approval	6 (5.4)
	tDCS training	26 (23.3)
	Patient consent	10 (8.9)
	I have no concerns	32 (28.6)
	Other	6 (5.4)
What are your concerns about the currently available therapy programs for the most common neurological conditions in your caseload?	Length of the treatment program	29 (25.9)
	Effectiveness of the treatment program	56 (50.0)
	Feasibility of the treatment program	25 (22.3)
What percentage of improvement would you need to see in your patients to implement tDCS as part of their therapy program?	Less than 20%	20 (17.9)
	20–30%	32 (28.6)
	31–50%	33 (29.5)
	More than 50%	27 (24.1)

## Data Availability

Due to privacy restrictions, the data presented in this study are available upon request from the corresponding authors.

## References

[1] Claflin E.S., Krishnan C., Khot S.P. (2015). Emerging treatments for motor rehabilitation after stroke. Neurohospitalist.

[2] Liew S.-L., Santarnecchi E., Buch E.R., Cohen L.G. (2014). Non-invasive brain stimulation in neurorehabilitation: Local and distant effects for motor recovery. Front. Hum. Neurosci..

[3] Boggio P.S., Nunes A., Rigonatti S.P., Nitsche M.A., Pascual-Leone A., Fregni F. (2007). Repeated sessions of noninvasive brain DC stimulation is associated with motor function improvement in stroke patients. Restor. Neurol. Neurosci..

[4] Stagg C.J., Nitsche M.A. (2011). Physiological basis of transcranial direct current stimulation. Neuroscientist.

[5] Albishi A.M. (2024). How does combining physical therapy with transcranial direct stimulation improve upper-limb motor functions in patients with stroke? A theory perspective. Ann. Med. Surg..

[6] Nitsche M.A., Cohen L.G., Wassermann E.M., Priori A., Lang N., Antal A., Paulus W., Hummel F., Boggio P.S., Fregni F. (2008). Transcranial direct current stimulation: State of the art 2008. Brain Stimul..

[7] Brunoni A.R., Amadera J., Berbel B., Volz M.S., Rizzerio B.G., Fregni F. (2011). A systematic review on reporting and assessment of adverse effects associated with transcranial direct current stimulation. Int. J. Neuropsychopharmacol..

[8] Elsner B., Kugler J., Pohl M., Mehrholz J. (2020). Transcranial direct current stimulation (tDCS) for improving activities of daily living, and physical and cognitive functioning, in people after stroke. Cochrane Database Syst. Rev..

[9] Page S.J., Cunningham D.A., Plow E., Blazak B. (2015). It takes two: Noninvasive brain stimulation combined with neurorehabilitation. Arch. Phys. Med. Rehabil..

[10] Rocha S., Silva E., Foerster Á., Wiesiolek C., Chagas A.P., Machado G., Baltar A., Monte-Silva K. (2016). The impact of transcranial direct current stimulation (tDCS) combined with modified constraint-induced movement therapy (mCIMT) on upper limb function in chronic stroke: A double-blind randomized controlled trial. Disabil. Rehabil..

[11] Marquez J., van Vliet P., McElduff P., Lagopoulos J., Parsons M. (2015). Transcranial direct current stimulation (tDCS): Does it have merit in stroke rehabilitation? A systematic review. Int. J. Stroke.

[12] Orru G., Baroni M., Cesari V., Conversano C., Hitchcott P.K., Gemignani A. (2019). The effect of single and repeated tDCS sessions on motor symptoms in Parkinson’s disease: A systematic review. Arch. Ital. De Biol..

[13] Pilloni G., Choi C., Shaw M.T., Coghe G., Krupp L., Moffat M., Cocco E., Pau M., Charvet L. (2020). Walking in multiple sclerosis improves with tDCS: A randomized, double-blind, sham-controlled study. Ann. Clin. Transl. Neurol..

[14] Inagawa T., Yokoi Y., Narita Z., Maruo K., Okazaki M., Nakagome K. (2019). Safety and feasibility of transcranial direct current stimulation for cognitive rehabilitation in patients with mild or major neurocognitive disorders: A randomized sham-controlled pilot study. Front. Hum. Neurosci..

[15] Brunoni A.R., Palm U. (2019). Transcranial direct current stimulation in psychiatry: Mood disorders, schizophrenia and other psychiatric diseases. Practical Guide to Transcranial Direct Current Stimulation: Principles, Procedures and Applications.

[16] Liao W.-W., Chiang W.-C., Lin K.-C., Wu C.-Y., Liu C.-T., Hsieh Y.-W., Lin Y.-C., Chen C.-L. (2020). Timing-dependent effects of transcranial direct current stimulation with mirror therapy on daily function and motor control in chronic stroke: A randomized controlled pilot study. J. Neuroeng. Rehabil..

[17] Abualait T.S. (2020). Effects of transcranial direct current stimulation of primary motor cortex on cortical sensory deficits and hand dexterity in a patient with stroke: A case study. J. Int. Med. Res..

[18] Bashir S., Al-Hussain F., Wasay M., Yoo W.-K. (2018). The effect of repetitive arm cycling training priming with transcranial direct current stimulation on post-stroke: Pilot study. Brain Neurorehabil..

[19] Pollock A., Farmer S.E., Brady M.C., Langhorne P., Mead G.E., Mehrholz J., Van Wijck F. (2014). Interventions for improving upper limb function after stroke. Cochrane Database Syst. Rev..

[20] Fregni F., Pascual-Leone A. (2007). Technology insight: Noninvasive brain sti- mulation in neurology—Perspectives on the therapeutic potential of rTMS and tDCS. Nat. Clin. Pract. Neurol..

[21] Ferreira I.S., Costa B.T., Ramos C.L., Lucena P., Thibaut A., Fregni F. (2019). Searching for the optimal tDCS target for motor rehabilitation. J. Neuroeng. Rehabil..

[22] Cho H.S., Cha H.G. (2015). Effect of mirror therapy with tDCS on functional recovery of the upper extremity of stroke patients. J. Phys. Ther. Sci..

[23] Elsner B., Kwakkel G., Kugler J., Mehrholz J. (2017). Transcranial direct current stimu- lation (tDCS) for improving capacity in activities and arm function after stroke: A network meta-analysis of randomised controlled trials. J. Neuroeng. Rehabil..

[24] O’Brien A.T., Bertolucci F., Torrealba-Acosta G., Huerta R., Fregni F., Thibaut A. (2018). Non-invasive brain stimulation for fine motor improvement after stroke: A meta-analysis. Eur. J. Neurol..

[25] Kim S.H. (2021). Effects of dual transcranial direct current stimulation and modified constraint-induced movement therapy to improve upper-limb function after stroke: A double-blinded, pilot randomized controlled trial. J. Stroke Cerebrovasc. Dis..

[26] Kang N., Summers J.J., Cauraugh J.H. (2016). Transcranial direct current stimu- lation facilitates motor learning post-stroke: A systematic review and meta-analysis. J. Neurol. Neurosurg. Psychiatry.

[27] Triccas L.T., Burridge J., Hughes A., Pickering R., Desikan M., Rothwell J., Verheyden G. (2016). Multiple sessions of tran- scranial direct current stimulation and upper extremity rehabilitation in stroke: A review and meta-analysis. Clin. Neurophysiol..

[28] Lefebvre S., Laloux P., Peeters A., Desfontaines P., Jamart J., Vandermeeren Y. (2013). Dual-tDCS enhances online motor skill learning and long-term retention in chronic stroke patients. Front. Hum. Neurosci..

[29] Bolognini N., Pascual-Leone A., Fregni F. (2009). Using non-invasive brain stimulation to augment motor training-induced plasticity. J. Neuroeng. Rehabil..

[30] Lefaucheur J.P., Wendling F. (2019). Mechanisms of action of tDCS: A brief and practical overview. Neurophysiol. Clin..

[31] Al-Hussain F., Nasim E., Iqbal M., Altwaijri N., Asim N., Yoo W.K., Bashir S. (2021). The effect of transcranial direct current stimulation combined with functional task training on motor recovery in stroke patients. Medicine.

[32] Bashir S., Aisha D., Hamza A., Al-Hussain F., Yoo W.K. (2021). Effects of transcranial direct current stimulation on cortex modulation by stimulation of the primary motor cortex and parietal cortex in humans. Int. J. Neurosci..

[33] Albishi A.M., Alhadlaq S.A., Altowairqi R.T., Alharbi M.F., Alsubiheen A.M., Alosaimi M.H., Bashir S., Alokaily A.O. (2024). Knowledge and attitude toward transcranial magnetic stimulation among rehabilitation specialists in Saudi Arabia. Front. Bioeng. Biotechnol..

[34] Alshehri M.M., Esht V., Alajam R.A., Alfifi A.H., Qahtani M.K., Alhwoaimel N., Alenazi A.M., Alqahtani B.A., Alhowimel A. (2024). Knowledge, Attitude, and Practices Among Clinical Physiotherapists Regarding Transcranial Direct Current Stimulation Application in Stroke Rehabilitation: Questionnaire Development and Validation via Multicenter Observations in Saudi Arabia. J. Disabil. Res..

[35] AlHadi A.N., AlShiban A.M., Alomar M.A., Aljadoa O.F., AlSayegh A.M., Jameel M.A. (2017). Knowledge of and attitude toward repetitive transcranial magnetic stimulation among psychiatrists in Saudi Arabia. J. ECT.

[36] Pourhoseingholi M.A., Vahedi M., Rahimzadeh M. (2013). Sample size calculation in medical studies. Gastroenterol. Hepatol. Bed Bench.

[37] Wasfi R.M., Alenezi F.S., Alzubaidi L.M., Hassanein M. (2024). The supply and demand for rehabilitation health workforce in Saudi Arabia. East. Mediterr. Health J..

[38] Yusoff M.S. (2019). ABC of content validation and content validity index calculation. Educ. Med. J..

[39] Terwee C.B., Bot S.D., de Boer M.R., Van der Windt D.A., Knol D.L., Dekker J., Bouter L.M., de Vet H.C. (2007). Quality criteria were proposed for measurement properties of health status questionnaires. J. Clin. Epidemiol..

[40] Albishi A.M. (2024). Knowledge, attitudes, and perceptions of physical therapists towards conventional physical therapy-across-sectional study. Ann. Med. Surg..

[41] Hussein H.M., Alshammari S.F., Alanazi I.A., Alenzy G.M., Alrashidy R.H. (2022). Sex-related differences in physical therapy career expectations in ha’il, Saudi Arabia. Acta Neuropsychol..

[42] Alghadir A., Zafar H., Iqbal Z.A., Anwer S. (2015). Physical therapy education in Saudi Arabia. J. Phys. Ther. Sci..

[43] Bindawas S.M. (2014). Physical therapy entry-level education and post-professional training in Saudi Arabia: A comparison of perceptions of physical therapists from five regions. J. Phys. Ther. Sci..

[44] Health M.O. (2013). Health Statistics Annual Book. https://www.moh.gov.sa/en/Ministry/Statistics/book/Documents/Statistics-Book-1434.pdf.

[45] Alrwaily M., Alanazi F. (2022). Prevalence and Determinants of Knowledge of Musculoskeletal Disorders Among Healthcare Providers and Students in Saudi Arabia: A Cross-Sectional Study. J. Multidiscip. Healthc..

[46] Al-Eisa E.S., Al-Hoqail H., Al-Rushud A.S., Al-Harthi A., Al-Mass B., Al-Harbi B.M., Al-Azzaz S., Alghadir A.H., Iqbal Z.A. (2016). Awareness, perceptions and beliefs about physiotherapy held by physicians working in Saudi Arabia: A cross-sectional study. J. Phys. Ther. Sci..

[47] Albishi A.M., Al-Ageel H.M., AlAbdulwahab S.S. (2024). Knowledge and Attitude Towards Bell’s Palsy Rehabilitation Among Physical Therapists in Saudi Arabia: A Cross-Sectional Study. Risk Manag. Healthc. Policy.

[48] Graham I.D., Logan J., Harrison M.B., Straus S.E., Tetroe J., Caswell W., Robinson N. (2006). Lost in knowledge translation: Time for a map?. J. Contin. Educ. Health Prof..

[49] Moncion K., Biasin L., Jagroop D., Bayley M., Danells C., Mansfield A., Salbach N.M., Inness E., Tang A. (2020). Barriers and facilitators to aerobic exercise implementation in stroke rehabilitation: A scoping review. J. Neurol. Phys. Ther..

[50] Vallejo P., Cueva E., Martínez-Lozada P., García-Ríos C.A., Miranda-Barros D.H., Leon-Rojas J.E., Cueva E.J. (2023). Repetitive transcranial magnetic stimulation in stroke: A literature review of the current role and controversies of neurorehabilitation through electromagnetic pulses. Cureus.

[51] Deng J., Gong Y., Lin X., Bao Y., Sun H., Lu L. (2020). Knowledge and attitudes about transcranial magnetic stimulation among psychiatrists in China. BMC Psychiatry.

[52] Bikson M., Grossman P., Thomas C., Zannou A.L., Jiang J., Adnan T., Mourdoukoutas A.P., Kronberg G., Truong D., Boggio P. (2016). Safety of transcranial direct current stimulation: Evidence based update 2016. Brain Stimul..

[53] Fregni F., Nitsche M.A., Loo C.K., Brunoni A.R., Marangolo P., Leite J., Carvalho S., Bolognini N., Caumo W., Paik N.J. (2015). Regulatory considerations for the clinical and research use of transcranial direct current stimulation (tDCS): Review and recommendations from an expert panel. Clin. Res. Regul. Aff..

[54] Cogan A.M., Weaver J.A., McHarg M., Leland N.E., Davidson L., Mallinson T. (2020). Association of length of stay, recovery rate, and therapy time per day with functional outcomes after hip fracture surgery. JAMA Netw. Open.

[55] Kumru H., Murillo N., Benito-Penalva J., Tormos J.M., Vidal J. (2016). Transcranial direct current stimulation is not effective in the motor strength and gait recovery following motor incomplete spinal cord injury during Lokomat(^®^) gait training. Neurosci. Lett..

